# Cone-beam computed tomography evaluation of periodontal and bone support loss in extraction cases

**DOI:** 10.1186/2196-1042-14-29

**Published:** 2013-09-11

**Authors:** Luca Lombardo, Romina Bragazzi, Carlo Perissinotto, Davide Mirabella, Giuseppe Siciliani

**Affiliations:** Postgraduate school of orthodontics, Ferrara University, Via Montebello 31, Ferrara, 44100 Italy; Via Masutto 11, Treviso, 31100 Italy

## Abstract

**Background:**

The aim of this study was to evaluate, in particular, whether bone resorption occurred at the extraction sites of a group of patients under orthodontic treatment, and, in general, whether extraction treatment predisposes patients to a greater degree of root resorption.

**Methods:**

The study group comprised 12 class II division 1 malocclusion patients who underwent orthodontic treatment and extraction, and the control group comprised 10 class II division 1 patients who underwent orthodontic treatment without extraction. In both groups, treatments were carried out by the same operator using the same techniques. Cone-beam computed tomography performed before (T1) and after (T2) treatment was used to determine and compare the root length, the distance from the cementoenamel junction to the base of the defect and to the bone peak, the width of the defect and the buccolingual bone thickness.

**Results:**

Root length was reduced following treatment in both groups, although to a statistically significantly greater extent in the study group. The buccolingual bone thickness was reduced after treatment in both groups, with no differences found between the study and control groups. The bone loss at the sites assessed was greater in the patients after extraction treatment, with a statistically significant difference revealed between the two groups. The site that showed the greatest variation in both groups was distal to the upper canines.

**Conclusions:**

In the present study, extractive orthodontic treatment appeared to predispose patients to a greater degree of root resorption. Indeed, the bone at the extraction site showed greater resorption in the study group with respect to the control group, and the appearance of intraosseous defects was noted in the former.

## Background

The effects that orthodontic forces have on the alveolar bone and on tooth roots have always been of great interest to researchers and clinicians alike [1–3]. Indeed, a considerable amount of attention has been focussed on the short- and long-term effects of orthodontic treatment, particularly since changes to the alveolar bone during dental movement have been reported, and the loss of crestal bone is a well-known feature of marginal periodontitis [4, 5]. The relationship between orthodontics and periodontics, in terms of orthodontic treatments and periodontal disease, has long provoked curiosity and interest, with researchers seeking to determine whether orthodontics and correct dental alignment are protective factors against periodontal disease or, conversely, whether orthodontic therapy is a factor that predisposes patients to problems of a periodontal nature [6–8]. This research has led to the recognition that an adequate periodontium, which provides sufficient root length and bone support, is a decisive factor with regard to the stability of orthodontic treatment outcomes. For this reason, it is fundamental that the interrelationships between orthodontic treatment, root resorption and modifications to the alveolar bone are studied in depth [9].

Although the literature has not yet furnished us with conclusive findings, clinical experience dictates that interproximal morphology is far from optimal in extraction cases. Orthodontic treatment plans often include dental extraction, in particular of the premolars, and it is this specific type of therapy that has been associated with changes in the architecture of the interdental bone spaces. It has been shown that the alterations to the gingival morphology following extraction can result in reductions in the interproximal bone levels and provoke the onset of root resorption [10–12]. The greater movement that the teeth must undergo and the resulting longer treatment times required in these cases may also be implicated.

Root resorption is a fairly common adverse effect of orthodontic treatment and has therefore attracted much attention of late, particularly in view of its medical legal ramifications [13, 14]. This increased attention has led Brezniak and Wasserstein to propose a new term for this type of resorption: orthodontically induced inflammatory root resorption [15]. In this context, the aim of the present study was to evaluate whether bone resorption occurs mesial and distal to the extraction site of teeth removed for orthodontic reasons in a group of patients under treatment, and to determine whether the extraction treatment predisposes patients to a greater degree of root resorption.

## Methods

To carry out this retrospective observational study, the pre-treatment cone-beam computed tomography (CBCT) radiographs were selected from the archives of a private orthodontic practice on the basis of the following inclusion criteria:

1. Class II division 1 malocclusion
2. Extraction of the upper first premolars and lower second premolars
3. Patients treated by means of the Tweed technique
4. Availability of good quality pre-treatment and post-treatment CBCT radiographs

The exclusion criteria were as follows:

1. The presence of agenesis
2. History of trauma or presence of dentoalveolar lesions
3. Previous orthodontic treatment

The pre-treatment and post-treatment records of 21 patients who underwent extraction as part of their orthodontic treatment were analysed, and of the 21 patients initially selected, 8 were excluded due to the absence of post-treatment CBCT radiographs and 1 for agenesis of the upper laterals. The final sample, defined as the study group, therefore comprised 12 patients (6 males, 6 females) with a mean age of 11 years and 9 months at the start of the treatment. All patients in the study group had undergone orthodontic treatment featuring extraction of the upper first premolars and the lower second premolars. The treatment was carried out by the same operator using the Tweed-Merrifield technique. The mean duration of treatment for the study group was 23.1 months.

The data collected for the study group were compared with those from a further experimental group, defined as the control group, with the aim of determining any significant differences between these two treatment groups. The control group comprised 10 patients (4 males, 6 females) who had undergone orthodontic treatment without extraction. This treatment was carried out by the same operator using the same technique. At the start of the treatment, the mean age of the control group was 10 years and 11 months, and the mean duration of treatment was 17.5 months.

For both of the treatment groups, measurements were obtained from the initial cone beams taken before the orthodontic treatment (T1) and then again from the radiographs taken immediately after the completion of treatment (T2). The reductions in root length and alveolar bone height were defined as the differences between T1 and T2. At T1, the ANB angle and overjet were measured on the cephalograms pertaining to both groups. The parameters of the two treatment groups are reported in Table 1.

**Table 1:** **Descriptive statistics of the two treatment groups**

Parameter	Study group^a^	Control group^a^
	(***n*** = 12)	(***n*** = 10)
Male/female ratio	1:1	2:3
ANB angle (°)	5.15 (±1.86)	4.76 (±0.93)
Overjet (mm)	4.61 (±2.05)	4.00 (±1.86)
Age at T1 (years)	11.75 (±1.42)	10.92 (±1.25)
Duration of treatment (months)	23.08 (±4.78)	17.50 (±3.02)

^a^Data are means (±SD), except for the ratio.

Data were obtained using a 3D volume scanner (QR Verona, NewTom 3G, Verona, Italy) based on a cone-beam technique that uses X-ray emissions efficiently, thereby reducing the dose absorbed by the patient. The following settings were used: a field of view of 12 in.; 110 kV (anteroposterior/laterolateral), 2.00 mA (anteroposterior) and 1.00 mA (laterolateral); and an exposure time of 5.4 s. The transverse sections on which the measurements were performed were extracted from the axial volume with the following characteristics:

- Width 50 mm
- Thickness 1 mm
- Step 0.5 mm

**Figure 1:**
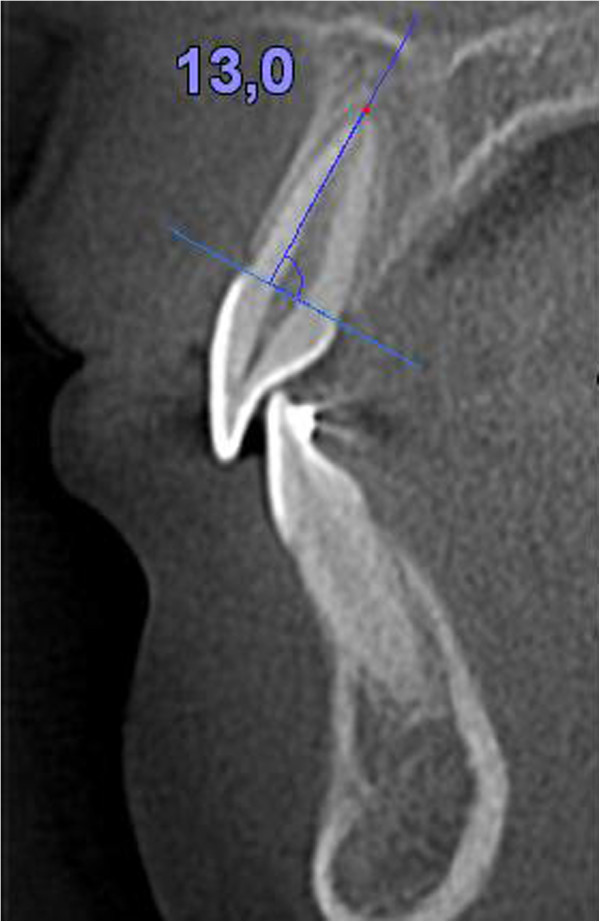
**Measurement of root length.**

**Figure 2:**
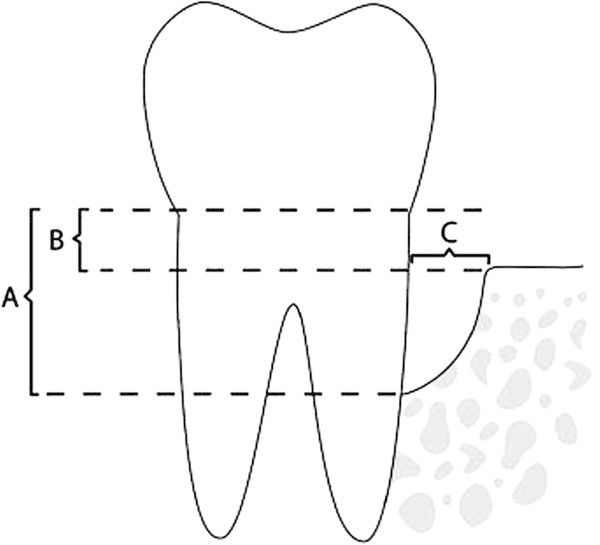
**Measurement of distances.** CEJ to defect base **(A)**, CEJ to bone peak **(B)** and defect width **(C)**. Accuracy of cone beam computed tomography for periodontal defect measurements [17].

**Figure 3:**
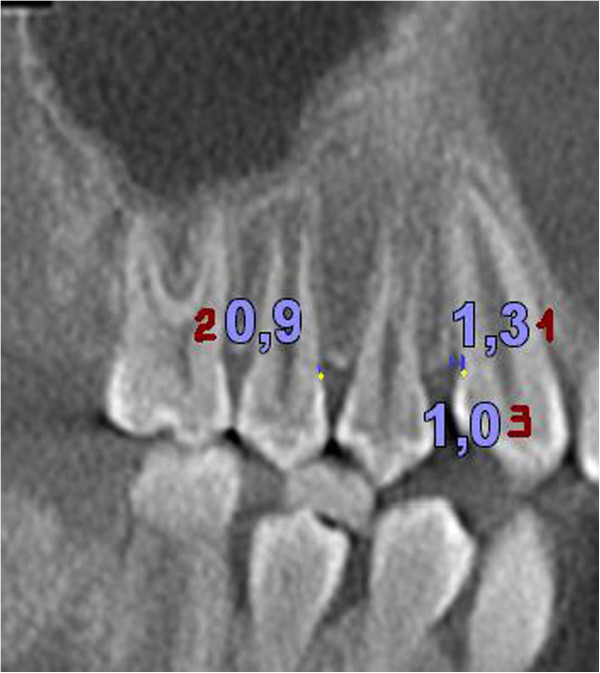
**Values obtained for the distances.** CEJ to defect base (1), CEJ to bone peak (2) and defect width (3).

**Figure 4:**
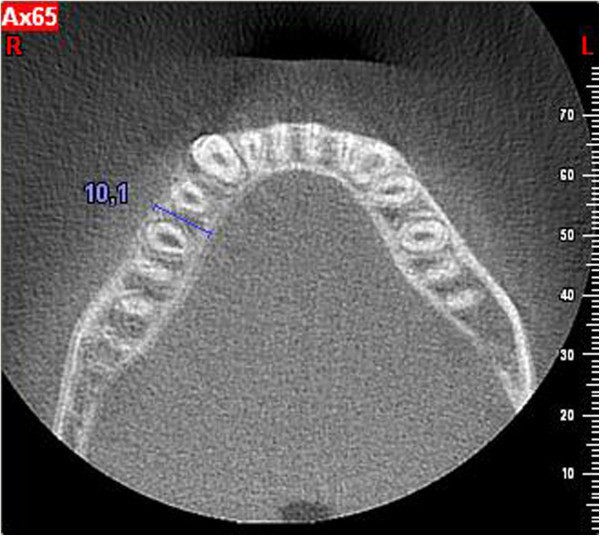
**Measurement of buccolingual thickness.**

Measurements were taken on a sagittal section passing through the root canal, along the long axis of the teeth [16]. From a line joining the mesial and distal cement-enamel junction (CEJ) of each dental element, the line perpendicular to the root apex was traced and used as a basis for the measurements (Figure 1). The root apex was positioned on the sagittal section, and the axial sections were then checked to confirm that the root apex was present in the last section before the root was no longer visible. To measure the alveolar bone at the extraction site, cross-sections passing through the fissure between the buccal and lingual cusps of the lower first premolars and upper second premolars were obtained, and four measurements were taken on each as follows (Figures 2, 3 and 4) [17]:

1. The distance from the CEJ to the base of the defect
2. The distance from the CEJ to the bone peak
3. The width of the defect
4. The buccolingual thickness of the bone

We excluded teeth with a rotation greater than 10°.

These measurements were taken for each extraction site at the following points:

1. The mesial surfaces of the left (25) and right (15) upper second premolars
2. The distal surfaces of the left (23) and right (13) upper canines
3. The mesial surfaces of the left (36) and right (46) lower first molars
4. The distal surfaces of the left (34) and right (44) lower first premolars

All measurements of root length and alveolar bone characteristics were repeated by the same operator after a maximum of 2 weeks so that the method error could be calculated; fifty measurements were taken - 10 for each type.

### Statistical analysis

For each study parameter, the means and standard deviations were calculated at the start and end of the treatments. Within each study group, the differences in the pre-treatment and post-treatment root lengths were analysed using Student's *t* test, based on comparisons between the means. In particular, given the make-up of the sample, the test applied considered the case of the mean of the differences between two dependent samples, i.e. as paired data. To evaluate whether there were any statistically significant differences between the root resorption measured in the study group and that in the control group, the Fisher *F* test was used, based on the comparison of variance. This test is designed to compare the internal variability of the groups with respect to the variability between the groups. When the first of these is relatively high, then the difference between the groups is probably due to internal variability alone.

To analyse the differences in the pre-treatment and post-treatment bone measurements within the groups (CEJ to defect base, CEJ to bone peak and defect width), Student's *t* test was again used. As the two groups do not have the same number of data items, the *t* test was used to analyse the means of the differences between two independent samples. Student's *t* test was also applied to the reduction in the buccolingual thickness, analysing the means of the differences of two dependent samples, as there was the same number of measures. To study the differences in the bone measurements between the two groups, the Fisher *F* test was used again. Finally, to determine the method error, Dahlberg’s formula, which defines the measurement error, was used. In particular, this equation gives the implicit level of difficulty in reading the measurements.

## Results

At T1, the root length and bone height of the two groups were comparable (Tables 2 and 3). The results obtained for the external apical root resorption are given in Table 4, and those for the bone measurements are given in Table 5. The changes in the root length (as a measure of the external apical root resorption) were recorded as the differences between the tooth lengths from T1 to T2 for the right (11) and left (21) upper central incisors, the right (12) and left (22) upper lateral incisors, the right (41) and left (31) lower central incisors, and the right (42) and left (32) lower lateral incisors. Analyses of the reduction in root length of the upper and lower incisors, according to Student's *t* test, showed statistically significant differences between the study and control groups from T1 to T2 (to a 95% probability level; Table 2). Thus, this test showed that the differences between the two groups were non-random and represented effects of the treatment itself. Only when the probability level was taken to 99% could it be shown that some of the differences might have been random, although only for the measurements relative to teeth 21 and 22 for the study group and for the measures relative to teeth 21, 31 and 41 for the control group. Analysis of the data through the Fisher *F* test showed that these differences between the study group and the control group were statistically significant. In particular, for the root resorption, the use of the Fisher *F* test verified that with a level of probability of 95%, the differences between the two groups were not random but were effects of the treatment.

**Table 2:** **Root length at T1 in the study and control groups**

Tooth ( ***N°*** )	Root length (mm)		***P*** value
	Study group	Control group	
	(***n*** = 12)	(***n*** = 10)	
Upper incisors			
Right central (11)	13.28	12.98	> 0.01
Right lateral (12)	13.31	12.85	> 0.01
Left central (21)	13	12.9	> 0.01
Left lateral (22)	12.94	12.93	> 0.01
Lower incisors			
Right central (41)	12.45	12.04	> 0.01
Right lateral (42)	13.54	12.43	> 0.01
Left central (31)	12.31	11.91	> 0.01
Left lateral (32)	13.05	12.46	> 0.01

**Table 3:** **Bone measurements at T1 in the study and control groups**

Bone parameter	Bone measurement		***P*** value
	Study group	Control group	
	(***n*** = 12)	(***n*** = 10)	
Lingual-vestibular bone thickness			
Maxillary	10.0	10.3	> 0.01
Mandibular	9.7	9.6	> 0.01
CEJ to the base of the defect			
Distal (13 23)	0.6	0,57	> 0.01
Mesial (15 25)	0.93	0.71	> 0.01
Distal (34 44)	0.98	0.91	> 0.01
Mesial (36 46)	0.97	1.06	> 0.01
CEJ to the bone peak			
Distal (13 23)	0.64	0.67	> 0.01
Mesial (15 25)	0.79	0.73	> 0.01
Distal (34 44)	0.88	0.95	> 0.01
Mesial (36 46)	0.96	0.96	> 0.01
Width of the defect			
Distal (13 23)	0	0	> 0.01
Mesial (15 25)	0.04	0	> 0.01
Distal (34 44)	0.03	0	> 0.01
Mesial (36 46)	0.04	0.09	> 0.01

**Table 4:** **Reductions in root length from T1 to T2 in the study and control groups**

Tooth ( ***N°*** )	Reduction in root length (T1-T2; mm)		***P*** value
	Study group	Control group	
	(***n*** = 12)	(***n*** = 10)	
Upper incisors			
Right central (11)	1.79	1.04	< 0.05
Right lateral (12)	1.51	0.83	< 0.05
Left central (21)	1.53	0.90	< 0.05
Left lateral (22)	1.28	0.96	< 0.05
Lower incisors			
Right central (41)	1.00	0.73	< 0.05
Right lateral (42)	1.46	0.70	< 0.05
Left central (31)	0.97	0.66	< 0.05
Left lateral (32)	1.37	0.77	< 0.05

Reductions in root length as the measure of the external apical root resorption.

**Table 5:** **Reductions in the bone measurements from T1 to T2 in the study and control groups**

Bone parameter	Bone measurement reductions (T1-T2; mm)		***P*** value*
	Study group	Control group	
	(***n*** = 12)	(***n*** = 10)	
Lingual-vestibular bone thickness			
Maxillary	1.71	0.67	> 0.1
Mandibular	3.19	0.98	> 0.1
CEJ to the base of the defect			
Distal (13 23)	1.08	0.25	< 0.05
Mesial (15 25)	0.58	0.10	< 0.05
Distal (34 44)	0.93	0.11	< 0.05
Mesial (36 46)	0.99	0.06	< 0.05
CEJ to the bone peak			
Distal (13 23)	0.54	0.17	< 0.05
Mesial (15 25)	0.49	0.24	< 0.05
Distal (34 44)	0.21	0.05	< 0.05
Mesial (36 46)	0.20	0.06	< 0.05
Width of the defect			
Distal (13 23)	0.42	0.16	< 0.05
Mesial (15 25)	0.22	0.12	< 0.05
Distal (34 44)	0.55	0.03	< 0.05
Mesial (36 46)	0.59	0.01	< 0.05

*P > 0.10, non-significant.

For the maxillary and mandibular bone thickness measurements, Student's *t* test showed that there were statistically significant differences between the study group and the control group in terms of the pre-treatment to post-treatment changes (to a 99% probability level; Table 3). However, analysis of this data through the Fisher *F* test revealed that the differences between the two groups were not significant. In particular, this test verified that the differences between the two groups in the apparent reduction in buccolingual thickness were not due to effects of the treatments.

For the remaining bone measurements, Student's *t* test revealed a statistically positive difference in the measurements carried out from the CEJ to the base of the bone defect, and the width of the bone defect in the study group (to a level of probability of 95%). However, in the measurements carried out from the CEJ to the bone peak, the test showed that this difference could be random. In the control group, the test did not reveal any statistically positive differences, in that it appears that the differences obtained in the various measurements were random. The Fisher *F* test applied to the differences between the two analysis groups showed that these were statistically significant. In particular, the test verified that in terms of the various measurements carried out on the base of the bone defect, the bone peak and the width of the bone defect, the differences in the two groups were due to an effect of the treatment, to a probability level of 95%. The greatest differences between the study group and the control group were seen for the measurements between the CEJ and the base of the defect and in the width of the defect.

The method errors evaluated through the Dahlberg formula are shown in Table 6.

**Table 6:** **Measurement error by parameter, according to the Dahlberg formula**

Parameter	Dahlberg parameter ***S*** error
Lingual-vestibular bone thickness	0.2992
CEJ to the base of the defect	0.1987
CEJ to the bone peak	0.1642
Width of the defect	0.1204
Root length	0.1817

## Discussion

The parameters for the study group and the control group at the start of the treatment were comparable. There were two values that showed the greatest differences here: the lengths of the treatments and the mean ages at the start of treatment. The length of the treatment, which was slightly longer in the study group (by 5.5 months), can be explained by the different types of treatment used; indeed, extraction treatment generally takes longer than its non-extractive counterpart. The older age in the study group (by almost 1 year), as well as being similar to that considered in other studies, can also be justified in that these patients are monitored until the best moment arrives for them to start treatment [18].

The error found in the method, quantified in our study through Dahlberg’s formula, is less than the values obtained in other studies in terms of root measurements [19]. The statistically significant reduction in root length found in the two groups is in agreement with data that have come from numerous other studies [10, 18, 20, 21]. The differences between the two groups in our study provide support for the hypothesis that, in the sample examined, the extraction was an important factor in the appearance of root resorption, a finding which contrasts with those of other studies in the literature [22–24]. Nevertheless, the average root resorption of the upper incisor was 1.53 ± 0.21 mm, which is in line with previous data. Parker et al. showed that for the upper central incisor, there was an average resorption of 1.40 mm in extraction patients, while Mirabella and Artun reported, in a group of adult patients, a resorption of 1.47 mm for the central incisor, 1.63 mm for the lateral and 1.25 mm for the canine [25, 26]. The values obtained in the present study are slightly greater, although not significant, and this could arise from the technique that we used to carry out the radicular measurements. Indeed, in comparison with periapical radiography and panoramic radiography, CBCT allows better identification of the CEJ, and it therefore consents more precise measurement of root length.

In contrast to the last two studies cited here, [25, 26] in our study, the tooth that showed the greatest level of resorption was the right central upper incisor, in both the study and control groups. In both of the groups, the mandibular teeth were resorbed to a lesser extent, which is in agreement with the majority of data found in the literature [27, 28]. The resorption that we found was on average less than 2 mm for each tooth. According to the literature, root resorption can vary from 1 to 2 mm for most orthodontic patients without any negative effects on the dental health or masticatory function [29]. In around 5% of adult patients and in 2% of adolescent patients under orthodontic treatment, the loss of radicular substance can be more than 5 mm, and resorption at this level can potentially undermine the longevity of the tooth [30]. In our study, only one patient showed a resorption that was this high, at the left upper lateral incisor, specifically 6.5 mm. Thus, even though the loss of root tissue seen in the study group (which was significantly greater with respect to the control group) is within the limit of root resorption normally seen after orthodontic treatment and is therefore of no real clinical concern, extraction treatment does appear to be associated with a higher level of root resorption.

In contrast to the frequency of studies on root resorption after orthodontic treatment, those focussing on reductions in the marginal bone heights are rare. Nevertheless, it is recognized that a loss of attachment can occur when the distance between the CEJ and the bone is greater than the mean values in the periodontally healthy population (i.e. when it is greater than 2 mm) [31, 32]. In our study group, the distance from the CEJ to the base of the defect varied between pre-treatment and post-treatment at all sites, with the lowest variation seen mesial to the upper second premolars. This can be explained by the fact that the entire upper extraction space was occupied by the canine in the cases analysed here, while the back teeth were maximally anchored. The resulting smaller movement of the second upper premolars might therefore explain the reduced bone resorption in this area. Furthermore, in terms of the measurements of defect width, the only value found to be not significant was at the site mesial to the second upper premolar.

As no differences were found in the measurement between the lower CEJ and the corresponding bone peak between T1 and T2 in the study group, it appears that in these cases, bone remodelling occurs mainly adjacent to the dental roots, while the bone peak tends towards less resorption, remaining almost stable, in particular in the lower arch. This inevitably results in intra-bone defects, with a consequent increase in the width of the defect, which was zero before the treatment in most patients (i.e. there is none), particularly in the lower arch.

Various studies have noted a greater loss of attachment in treated (with or without extraction) with respect to untreated patients [2, 3, 33]. Even though, on average, the distances between the CEJ and the defect base and between the CEJ and the bone peak are under the critical 2 mm in the study group, when these were greater than 2 mm, loss of attachment did occur at the sites under investigation, i.e. around 20% of cases. Loss of attachment was documented in no cases from the control, i.e. non-extractive, group. The mean loss of attachment in our study group (the distance from the CEJ to the base of the defect) was 0.9 mm in the teeth adjacent to the extraction site. The differences found in the study group do not occur in the control group, and indeed, in the control group, the only value that varies to a statistically significant level between pre-treatment and post-treatment is the distance between the CEJ and the base of the defect distal to the upper canines. This site is the site that has the highest level of resorption in both of groups in our study, similar to data reported by Zachrisson and Alnaes [3].

The statistically significant differences found between the study group and the control group for each site analysed appear to support the hypothesis that the bone corresponding to the extraction site undergoes remodelling and that extractive orthodontic treatment influences bone resorption to a greater extent than non-extractive orthodontic treatment. When evaluating the data collected here, we need to take into account two factors. The first is that CBCT allows a more precise three-dimensional examination of the defect with respect to traditional techniques, but unlike periapical radiography, it does tend to overestimate the defect. When the bone thickness is less 0.4 mm, the size of the acquisition voxel, the height of the bone, is underestimated. A reduction in the voxel from 0.4 to 0.25 mm improves the accuracy of the measurement but results in an increase in the radiogenic dose to the patient, and it is preferable therefore to avoid this in normal clinical practice [34]. The second factor to bear in mind is that in this study, the analyses were carried out immediately after treatment, and follow-up images were not taken into account. We cannot therefore state whether or not the defects undergo spontaneous resolution over time. Nonetheless, many long-term studies do seem to indicate that there are no differences between treated and untreated patients several years down the line [4, 5, 11, 35].

## Conclusions

The present study reveals the following:

1. There is a reduction in root length after orthodontic treatment, whether extractive or not.
2. There are statistically significant differences between the study group and the control group in the degree of root resorption, even though the resorption seen in the study group remained within clinically acceptable limits.
3. Both extractive and non-extractive orthodontic treatments resulted in a reduction in the buccolingual thickness of the alveolar bone, without, however, any statistically significant differences between the two.
4. Extractive orthodontic treatment did appear to exert an influence on bone remodelling, as an increase in the distance from the CEJ to the base of the defect and the appearance of infra-osseous defects were seen, in particular in the lower arch.

Nevertheless, we are not in a position to determine whether resolution of these defects occurs in the long term once the applied forces are removed and the orthodontic movement has ceased.
